# Exploring the efficacy of traditional Chinese medicine exercise in alleviating anxiety and depression in older adults: a comprehensive study with randomized controlled trial and network meta-analysis

**DOI:** 10.3389/fpsyg.2023.1290471

**Published:** 2023-12-11

**Authors:** Yangjian Dong, Xiaoqin Kuang, Lili Dong, Guodong Chao, Juancai Qi, Xinxin Zhang, Jiwei Yao

**Affiliations:** ^1^College of Physical Education and Health, Guangxi Normal University, Guilin, China; ^2^College of Physical Education, China Three Gorges University, Yichang, China; ^3^College of Physical Education and Health, Guangxi University, Guilin, China; ^4^School of Physical Education, Wuhan University of Technology, Wuhan, China

**Keywords:** traditional Chinese medicine exercise, anxiety, depression, older adults, network meta-analysis

## Abstract

**Background:**

Anxiety and depression pose a significant global health challenge for elderly individuals. Research has demonstrated the potential of traditional Chinese medicine (TCM) exercise therapies in alleviating these conditions. However, ongoing debate and uncertainty persist regarding the optimal therapy and its impact on anxiety and depression. This study aims to evaluate and prioritize TCM exercise therapies for anxiety and depression in older adults, to identify the most effective intervention, and to provide a basis for informed decision-making in clinical practice.

**Methods:**

We conducted a comprehensive search of electronic databases including The Web of Science, PubMed, the Cochrane Library, China National Knowledge Infrastructure (CNKI), Wang Fang, and Wei Pu database up to July 2022. Two researchers independently reviewed all included studies and extracted relevant data. Traditional meta-analysis was performed using Review Manager version 5.4, while network meta-analysis was conducted using STATA software version 15.1 to generate network evidence plots and funnel plots.

**Result:**

A total of 30 trials, involving 2,806 participants, met the eligibility criteria. The traditional meta-analysis revealed that TCM exercise significantly improved anxiety (SMD = −0.82, 95% CI = −1.39, −0.26, *p* = 0.004) and depression (SMD = −0.63, 95% CI = −0.85, −0.41, *p* < 0.01) compared to the control group. In the network meta-analysis, Tai Chi exercise was ranked as the most effective intervention for anxiety (68.3%), followed by Yi Jin Jing (63.6%). For depression, the Tai Chi exercise was ranked as the most effective (87.8%), followed by the Ba Duan Jin exercise (74.1%).

**Conclusion:**

TCE exercise can improve anxiety and depression in older adults, Among the four TCE exercise therapies included, Tai Chi exercise showed better efficacy than other types of treatment. Nevertheless, further research is required to validate the effectiveness of this exercise therapy through larger and more rigorous clinical trials.

**Systematic review registration:**

http://www.crd.york.ac.uk/PROSPERO/, identifier CRD42023438697.

## Introduction

Anxiety and depression have been identified by the World Health Organization as prevalent mental illnesses, significant contributors to disability and mortality, and major factors in the global economic burden (Krause et al., 2021; Zenebe et al., 2021). The prevalence of anxiety and depression is influenced by political, economic, and social factors. These psychological issues are experiencing a global increase, with an estimated 18.4% rise in depression and a 14.9% increase in anxiety from 2005 to 2015 (Hay et al., 2017). Moreover, there has been a notable surge in the prevalence of anxiety and depression amidst the COVID-19 pandemic over the past 3 years (Peng et al., 2020; Santomauro et al., 2021). Disparities in access to mental health services are pronounced on a global scale, particularly in low- and middle-income countries, where patients have limited access to services compared to those in developed countries due to the inadequate availability of mental health resources (Jaeschke et al., 2021).

Due to various factors such as age, physiological changes, and lifestyle modifications, older adults are susceptible to insufficient intake of trace elements, which in turn can affect their cognitive abilities and physical fitness levels (Akbari et al., 2022). Reduced cognitive function and physical weakness often lead to psychological issues such as anxiety and depression in older adults, which may trigger other chronic diseases. Currently, drug therapy is the primary treatment option for most patients (Wu et al., 2017). However, drug therapy is often limited by poor efficacy and side effects, which are particularly severe in older adult populations. Moreover, drug therapy increases the cost of mental health care, placing unnecessary burdens on patients and their families, thereby negatively impacting their quality of life. In contrast, psychological treatment interventions can effectively improve anxiety and depression (Faraji et al., 2021a,b). However, psychological treatment requires face-to-face interventions between patients and therapists, and many patients refuse psychological treatment interventions due to the stigma associated with their symptoms. Furthermore, psychological treatment is often expensive. Therefore, it is crucial to choose low-cost and side-effect-free alternative treatments. Research has shown that physical exercise can improve participants’ mental health and increase their cognitive levels (Taheri et al., 2018; Muhammad, 2020; Soylu et al., 2022). However, the motivation to participate in sports activities also affects the timeliness of exercise adherence (Silva et al., 2022). Due to the high intensity and complex skills required in many sports activities, these factors make it challenging to stimulate the motivation of older adults to engage in physical activities, making it difficult for them to persist.

As an essential component of non-pharmacological treatment, Traditional Chinese Medicine (TCM) exercise has been widely utilized in the treatment of anxiety and depression due to its site limitations-free and adverse effect-free characteristics and has achieved good clinical therapeutic effects. TCM exercise therapies such as Tai Chi and Qigong (Ba Duan Jin, Wu Qin Xi, Yi Jin Jing, Liu Zi Jue) have been shown to improve cardiovascular function (Cassiano et al., 2020), blood circulation (Liang et al., 2019), cognitive function (Liu et al., 2021), and sleep quality. In recent years, there has been a global increase in the widespread adoption of TCM exercise. Meanwhile, a systematic review and meta-analysis have demonstrated that TCM exercise can alleviate anxiety and depression in middle-aged and older adult populations.

However, these TCM exercises vary in form, duration, and population, and it is not clear which therapy is more appropriate for older adults with the disease. In addition, most RCTs only compared non-pharmacological and conventional treatments, the non-exercise group and the waitlist, and few RCTs compared the effects of interventions between Tai Chi and Qigong. Therefore, a traditional systematic review to assess the effectiveness of interventions across treatment modalities is required. Network Meta-analysis (NMA) is a technique used to compare different interventions (Lei et al., 2022). NMA can compare multiple interventions directly and indirectly and select the best interventions based on the evidence from the network (Liu et al., 2022). Consequently, in this study, we used reticulated meta-analysis combined with clinical trial data and evaluated the interventions, ranking the interventions according to the results and eliminating the less effective ones. The purpose of this study is to provide the best treatment choice for patients with anxiety and depression and to inform clinicians when choosing non-pharmacological treatment decisions.

## Materials and methods

This systematic review and network meta-analysis was registered (identifier: CRD42023438697) in the International Prospective Register of Systematic Reviews (PROSPERO) and strictly followed the Preferred Reporting Items for Systematic Review and Meta-analysis for Network Meta-analysis (PRISMA-NMA) statement (Su et al., 2022). Due to this being a systematic review, there was no need the Ethics approval.

### Study search and selection

The following six electronic databases were searched up to July 2023. It includes the Cochrane Central Register of Controlled Trials, Web of Science, PubMed, China National Knowledge Infrastructure (CNKI), Chinese Wan Fang and Wei Pu database, and was Restricted languages are English and Chinese. We used various medical subject headings and free terms for the search, including but not limited to traditional Chinese medicine exercise, traditional Chinese exercise, Tai Chi, Wu Qin Xi, Ba Duan Jin, Yi Jin Jing, Liu Zi Jue, mind–body exercise, older adults, anxiety, depression, and randomized controlled trials. In addition, we also searched the reference lists of included articles to identify studies that met our criteria. The specific search strategies are detailed in the appendix.

Inclusion criteria: (a) older adults aged ≥55 years, (b) the experimental group intervention measures using Tai Chi, Qigong (Ba Duan Jin, Wu Qin Xi, Yi Jin Jing, Liu Zi Jue), the control group includes health care, routine nursing, or other therapies different from the experimental group intervention, (c) The research report indicates that anxiety or depression is one of the outcome measures. Standardized scales for measuring anxiety or depression indicators must be used. The results must be recorded immediately before and after treatment, and if more than two different scales are used to measure the same indicator, the pre-and post-test values of the objective measurement scale should be adopted. (d) all types of studies are randomized controlled trials.

Exclusion criteria: (1) intervention methods that are unclear or mixed with other exercises; (2) studies without analyzable data, such as studies that do not report values (i.e., mean, standard deviation, and sample size) used to calculate effect size; (3) unclear description of participant age.

To eliminate duplicate entries from the search results, the EndNote X9 software was employed. Then, two reviewers conducted an independent assessment of the titles and abstracts of the articles to ascertain their suitability for inclusion in the study. Further review was not conducted for studies that did not meet the inclusion criteria. Unexcluded studies were assessed by two reviewers (YD and XK) Full text. Discrepancies or uncertainties were addressed through deliberation including a third assessor (JY).

### Data extraction and quality assessment

Two reviewers independently extracted data from randomized controlled trials according to standardized forms, including (the name of the first author, the year of publication, the sample size, gender, age, type of traditional Chinese medicine intervention, control intervention, intervention frequency, intervention duration, primary outcome measures, and measurement tools and Indicators for pre- and post-testing). In the event of any ambiguous or insufficient information in the study, it is imperative to reach out to the first author for clarification and to acquire the necessary details. This study used the Cochrane 5.1.0 version bias risk tool to assess the quality of the included randomized controlled trials. The assessment criteria covered seven items, namely random sequence generation, allocation concealment, blinding of participants and personnel, blinding of outcome assessors, incomplete outcome data, selective reporting, and other biases. Each item was classified as high risk, low risk, or unclear risk of bias. The criteria for judgment were as follows: if random sequence generation and allocation concealment were not stated, it was considered high risk; considering the special nature of sports intervention, if blinding was not implemented during the intervention and blinding was not used for outcome assessment, it was considered unclear risk; if loss to follow-up and data exclusion were not reported, it was considered high risk, and if not mentioned, it was considered unclear risk; if there was no selective reporting, it was considered low risk; if there were no other biases that could cause risk, it was considered low risk. Two reviewers (DY and KX) completed the assessment work. If there were differences in the evaluation, the two reviewers would discuss the differences, and if the differences could not be resolved, the third reviewer would be consulted and make the final decision.

### Data synthesis and analysis

Firstly, we performed a meta-analysis using Review Manager 5.4. Considering that the outcome measure was a continuous variable and there were significant differences in the scoring tools for anxiety and depression measures among the articles, we used the standard deviation (SMDs) and 95% CI as the effect measures. When *p* < 0.05, it was considered statistically significant. In the heterogeneity test, we used the I^2^ test. If the heterogeneity was small (*p* < 0.1, I^2^ < 50%), a fixed-effects model was used. If the heterogeneity was large (*p* > 0.1, I^2^ > 50%), a random-effects model was used, and subgroup analysis was conducted to explore the source of heterogeneity (Borenstein et al., 2010).

We performed network meta-analysis using Stata 15.1 software and conducted inconsistency tests using node analysis (Shim et al., 2017). If *p* > 0.05, it indicates that there was no statistically significant difference between direct and indirect comparisons, and the two results were consistent, so a consistency model analysis was used. Otherwise, an inconsistency model was used. By comparing the cumulative probability plot area and the area under the curve, the ranking of interventions was determined. A higher SUCRA value indicated a better effect of the intervention (Salanti et al., 2011). In addition, to evaluate the robustness of the research results and determine whether the results were influenced by the characteristics of the studies, sensitivity analysis was conducted based on the baseline characteristics of the patients. After excluding studies involving non-Chinese populations and studies using physical exercise as the control group, new meta-analyses were performed, and the combined effect sizes remained within the 95% confidence interval, indicating that the research results were robust. To evaluate publication bias, we examined the funnel plot for potential signs of bias. If the effect sizes of the studies included in the analysis were evenly distributed, this suggests minimal publication bias in the network meta-analysis.

## Results

### Study inclusion and selection

**Figure 1:**
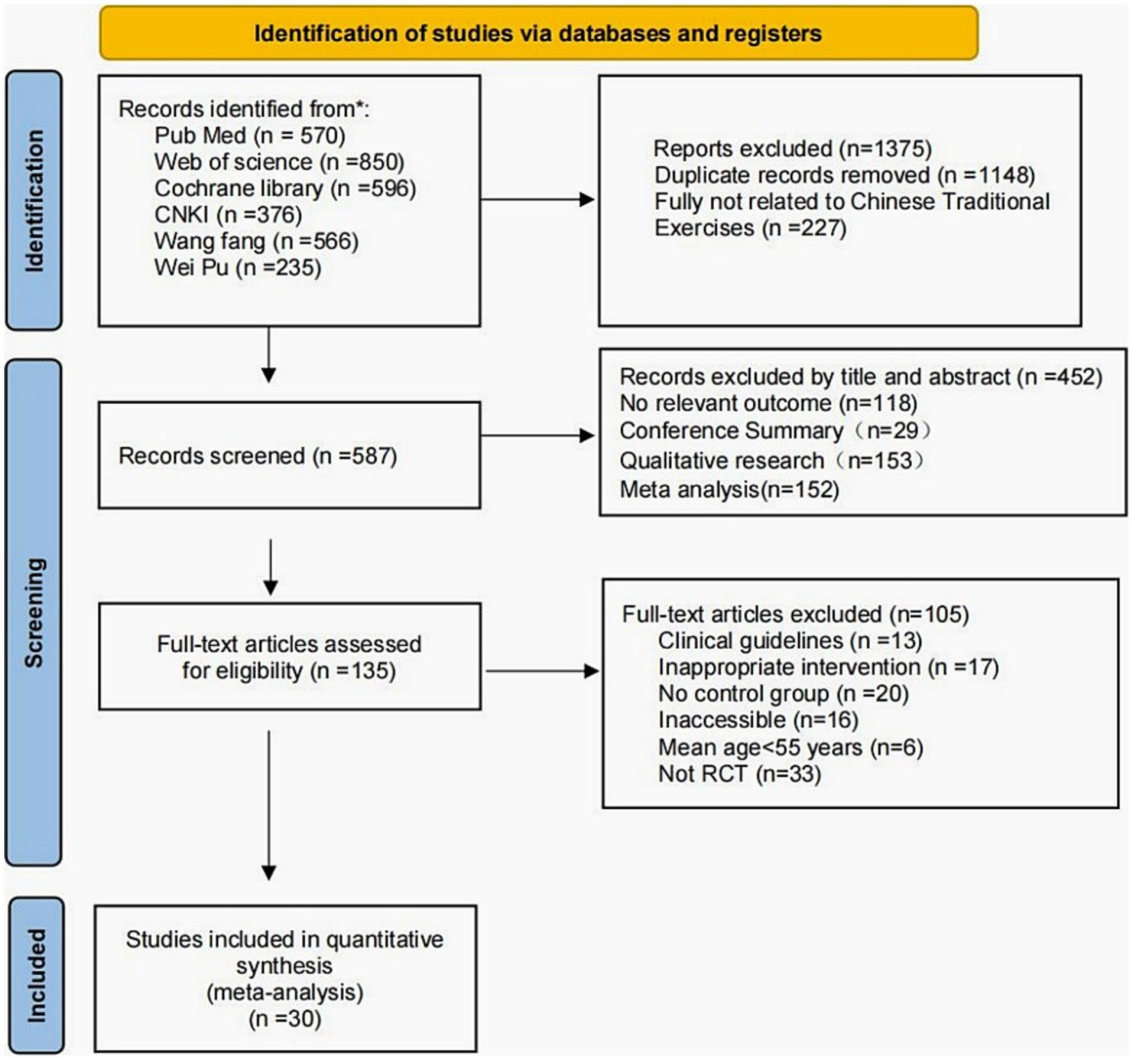
The flow of literature search and selection process.

Through a systematic search of 6 databases, a total of 30 RCTs involving 2,806 participants were included, and the methods for literature screening were described. After removing duplicate articles, a total of 587 articles were included through title and abstract screening, while a total of 452 articles were excluded from the analysis as they did not meet the inclusion criteria. A comprehensive review of the remaining 153 articles resulted in the exclusion of 104 irrelevant studies. This included 13 reports that were clinical guidelines, 16 articles that were inaccessible, 17 articles with inappropriate intervention, 20 articles with no control group, 6 articles with mean age < 55 years, and 33 articles not RCTs. Finally, this network meta-analysis included 30 published randomized controlled trials, involving a total of 2,806 participants, comparing the effects of Tai Chi, Qigong (Ba Duan Jin, Yi Jin Jing, Liu Zi Jue), and control groups on improving anxiety and depression in older adults. These studies, mostly from China, were published between 2011 and 2021. The interventions in the included trials mainly consisted of Tai Chi and Qigong (Ba Duan Jin, Yi Jin Jing, Liu Zi Jue). Most trials used routine care, education, or other non-aerobic exercises as controls. The duration of each intervention varied from 20 to 120 min per session, and the frequency ranged from 1 to 7 times per week. In these 30 randomized controlled trials, the majority of anxiety assessments were conducted using the Generalized Anxiety Scale (GAS), while depression assessments were done using the Geriatric Depression Scale (GDS). Figure 1 depicts the process of study selection for eligible studies, while Table 1 presents the characteristics of the studies included in this network meta-analysis.

**Table 1:** Detailed characteristics of each of the included literature studies.

Study	Country	Sample size		Mean age years		Intervention		Frequency and period	Outcome measures	Outcome
		E	C	E	C	E	C
Bonab and Parvaneh (2022)	Iran	35	35	60.00–70.00		Tai Chi	Routine daily activities	Three 60-min sessions/12 weeks	DAS	Anxiety
Liu et al. (2018)	China	30	30	60.90 ± 4.28	61.72 ± 3.54	Tai Chi	Routine daily activities	Three 60-min sessions/24 weeks	GDS	Depression
Liao et al. (2018)	China	55	52	71.80 ± 7.29	71.75 ± 8.20	Tai Chi	Health education	Three 50 min sessions/3 months	GDS	Depression
Ge et al. (2022)	China	32	33	70.16 ± 5.40	72.91 ± 6.61	Tai Chi	Routine daily activities	Three 60-min sessions/8 weeks	GDS	Depression
Song et al. (2022)	China	20	20	64.15 ± 8.56		Tai Chi	Health education	Three 60-min sessions/12 weeks	SAS.SDS	Anxiety depression
Li et al. (2019)	China	163	163	63.61 ± 6.62	65.44 ± 5.79	Tai Chi e	Physical exercise	Seven 60-min sessions/6 months	SAS.SDS	Anxiety depression
Redwine et al. (2020)	USA	24	23	63.00 ± 9.00	67.00 ± 7.00	Tai Chi	Usual care	Two 60-min sessions/16 weeks	BDI-IA	Depression
Lam et al. (2014)	China	171	218	77.20 ± 6.30	78.30 ± 6.60	Tai Chi	Physical exercise	Three 30-min sessions/1 year	CSDD	Depression
Hsu et al. (2016)	China	30	30	80.70 ± 9.68	81.77 ± 6.32	Tai Chi	Routine daily activities	Three 40 min sessions /26 weeks	GDS	Depression
Chou et al. (2004)	China	7	7	72.60 ± 4.20		Tai Chi	Usual care	Three 45 min sessions/3 months	CES-D	Depression
Ma et al. (2018)	China	79	79	70.20 ± 10.25	69.70 ± 10.84	Tai Chi	Usual care	Three to five 90 sessions/6 months	CES-D	Depression
Leung et al. (2013)	Australia	19	19	73.00 ± 8.00		Tai Chi	Usual care	Five 30 min sessions/12 weeks	HADS	Anxiety depression
Yeh et al. (2020)	American	61	31	68.60 ± 9.20	68.10 ± 6.70	Tai Chi	Health education	Three 30 min sessions/12 weeks	CES-D	Depression
Yildirim et al. (2016)	Turkey	30	30	62.90 ± 6.50	64.40 ± 7.50	Tai Chi	Physical exercise	Three 1 h sessions/12 weeks	GDS	Depression
Solianik et al. (2021)	Lithuania	15	15	≥60.00		Tai Chi	Usual care	Two 60 min sessions/10 weeks	HADS	Anxiety depression
Noradechanunt et al. (2017)	Australia	13	13	66.60 ± 6.70		Tai Chi	Physical exercise	Two 90 min sessions/24 weeks	CES-D	Depression
Lavretsky et al. (2011)	Turkey	36	37	69.10 ± 7.00	70.00 ± 7.40	Tai Chi	Health education	One 2 h sessions/10 weeks	HAMA.HAMD	Anxiety depression
Irwin et al. (2014)	USA	48	25	66.30 ± 7.40	66.40 ± 7.70	Tai Chi	Health education	120 min/week for 4 months	IDS-C	Depression
Tsang et al. (2013)	China	21	17	79.67 ± 6.55	80.65 ± 4.36	Ba Duan Jin	Usual care	Three 45-min sessions/12 weeks	HRSD	Depression
Gao (2016)	China	45	42	57.20 ± 8.70	56.70 ± 9.00	Liu Zi Jue	Routine daily activities	Five to seven 30 min sessions/3 months	HAMA	Anxiety
Zhong et al. (2006)	China	109	94	63.17 ± 5.80	62.06 ± 6.10	Yi Jin Jing	Usual care	Five 1 h sessions/1 year	SCL-90	Anxiety depression
Sun et al. (2022)	China	30	30	62.03 ± 7.37	65.23 ± 6.29	Yi Jin Jing	Routine daily activities	Seven 40 min sessions/3 weeks	HAMD	Depression
Wei (2013)	China	80	88	69. 40 ± 7. 10	70. 90 ± 7.80	Ba Duan Jin	Usual care	Six to seven 1 h sessions/5 months	SCL-90	Anxiety depression
Kan (2008)	China	35	30	≥60.00		Tai Chi	Routine daily activities	Seven 30 min sessions/6 months	CMI	Anxiety depression
Yuan et al. (2016)	China	30		66.33 ± 5.56	67.47 ± 3.82	Tai Chi	Usual care	Five 20–40 min sessions/12 weeks	HAMD	Depression
Liu (2016)	China	32	31	66.30 ± 2.70	65.80 ± 3.20	Tai Chi	Usual care	Five 60 min sessions /16 weeks	SCL-90	Anxiety depression
Liao (2015)	China	40	40	63. 10 ± 4. 20	62. 80 ± 3. 65	Tai Chi	Usual care	Three 60 min sessions /24 weeks	SCL-90	Anxiety depression
Hong et al. (2017)	China	60	60	≥60.00		Tai Chi	Routine daily activities	Four 60 min sessions /8 weeks	SCL-90	Anxiety depression
Li et al. (2017)	China	30	30	65.83 ± 5.71	56.27 ± 5.40	Tai Chi	Routine daily activities	Five to six 80 min sessions /6 months	HAMA	Anxiety
Huang et al. (2019)	China	36	38	81.9 ± 6.0	81.9 ± 6.1	Tai Chi	Usual care	Three 20 min sessions/10 months	GDS	depression

E, Experiment group; C, control group; DAS, death anxiety scale; GDS, Geriatric Depression Scale; SAS, Self-rating Anxiety Scale; SDS, Self-rating depression Scale; BDI-IA, The 21-item Beck Depression Inventory − 1A; CSDD, Cornell Scale for Depression in Dementia; CES-D, Center for epidemiologic studies depression scale; HADS, Hospital Anxiety and Depression Scale; IDS-C, Inventory of Depressive Symptomatology; HRSD, Hamilton Rating Scale of Depression; HAMA, Hamilton Rating Scale of Anxiety; HAMD, Hamilton Rating Scale of Depression; CMI, Cornell Medical Index.

### Assessment of risk of bias and quality of studies

**Figure 2:**
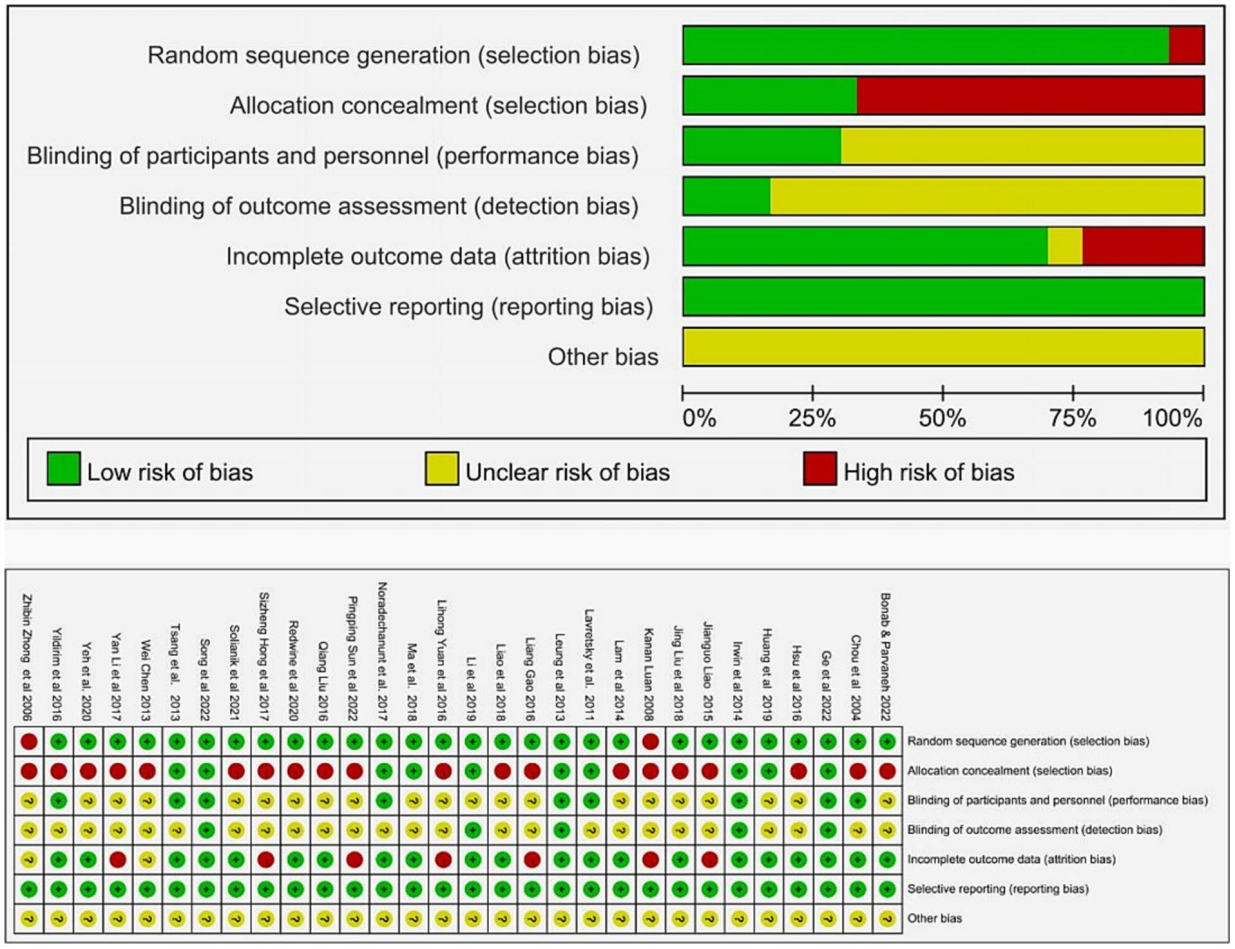
Quality assessment of included studies.

The quality of the selected studies was evaluated using the Cochrane risk-of-bias tool, and the findings are presented in Figure 2. The majority of the trials (94%) included in the analysis presented a description of random allocation and exhibited a minimal risk in terms of randomized allocation. Among the RCTs included, 11 (52%) offered a concise account of the random sequence generation process, utilizing either a computer or a random number table. The majority of trials had unclear allocation hidden risks, and only 11 trials (35%) with a single-blind design had a low risk of performance bias. Due to exercise being the intervention, a limited number of studies (26%) implemented blinding techniques for participants and personnel. A total of 25 studies (81%) showed an unclear assessment of outcome blindness. A total of 24 studies (77%) provided detailed explanations for the loss of follow-up during the experimental process. Only six studies (19%) provided the clinical registration numbers for the experiments.

### Traditional meta-analysis

**Figure 3:**
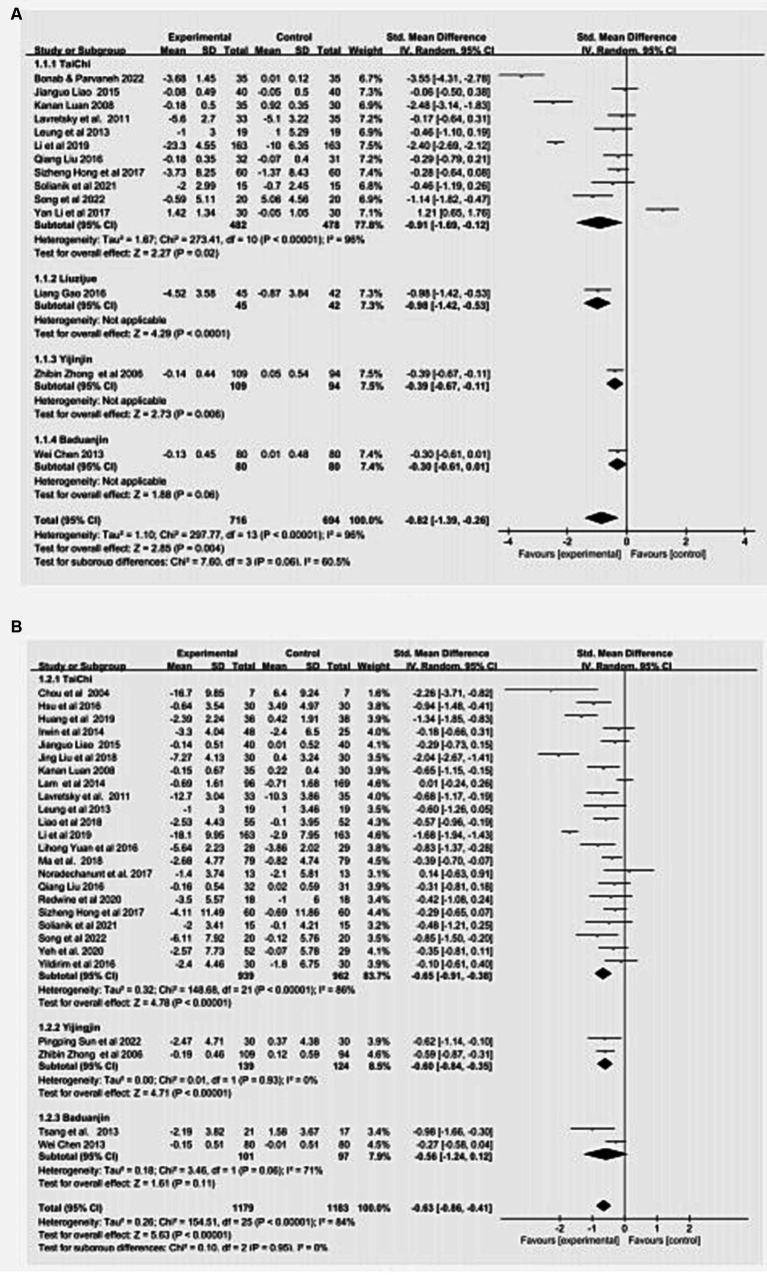
Pairwise meta-analysis of TCM Exercise on anxiety **(A)** and depression **(B)**. Meta-analysis results for pair-wise comparisons represented by SMD and 95% credible interval.

We conducted a traditional meta-analysis to compare the effects of TCM exercise interventions with non-TCM exercise interventions, which include physical exercise, routine care, daily activities, and health education. The results indicated that TCM exercise significantly reduced anxiety (SMD = −0.82, 95% CI = −1.39, −0.26, *p* = 0.004) and depression (SMD = −0.63, 95% CI = −0.85, −0.41, *p* < 0.01) compared to non-TCM exercise therapy. The findings of the traditional meta-analysis can be seen in Figure 3.

### Network meta-analysis

**Figure 4:**
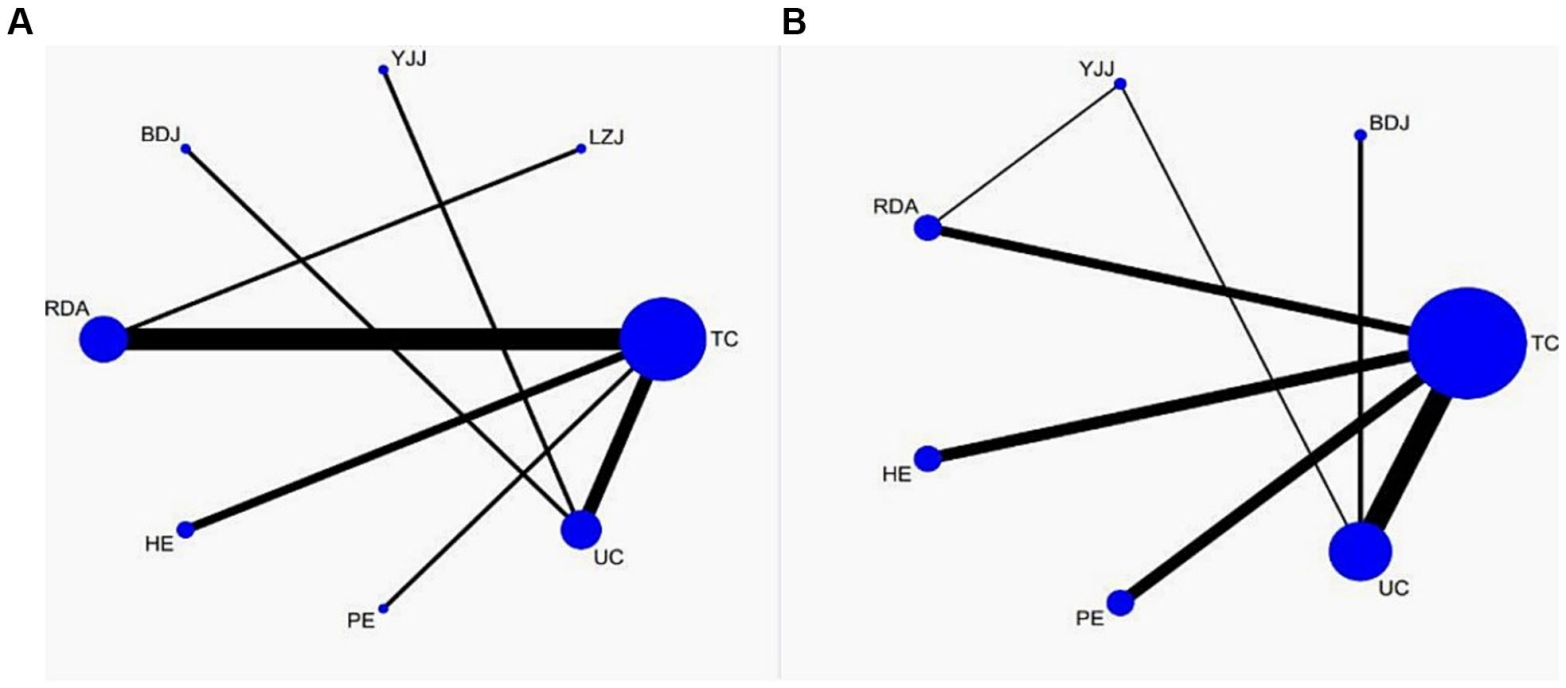
The network structure of the analyzed treatment comparisons for the outcome of anxiety **(A)** and depression **(B)**.

We performed a network meta-analysis to evaluate and rank the comparative impacts of different interventions on anxiety. The network diagram in Figure 4A displays 14 studies and 8 interventions related to anxiety. The node splitting analysis indicated a value of *p* greater than 0.05 and a PSRF value of 1, indicating model convergence and relatively robust results. Therefore, we chose the consistency model for the subsequent network analysis.

**Figure 5:**
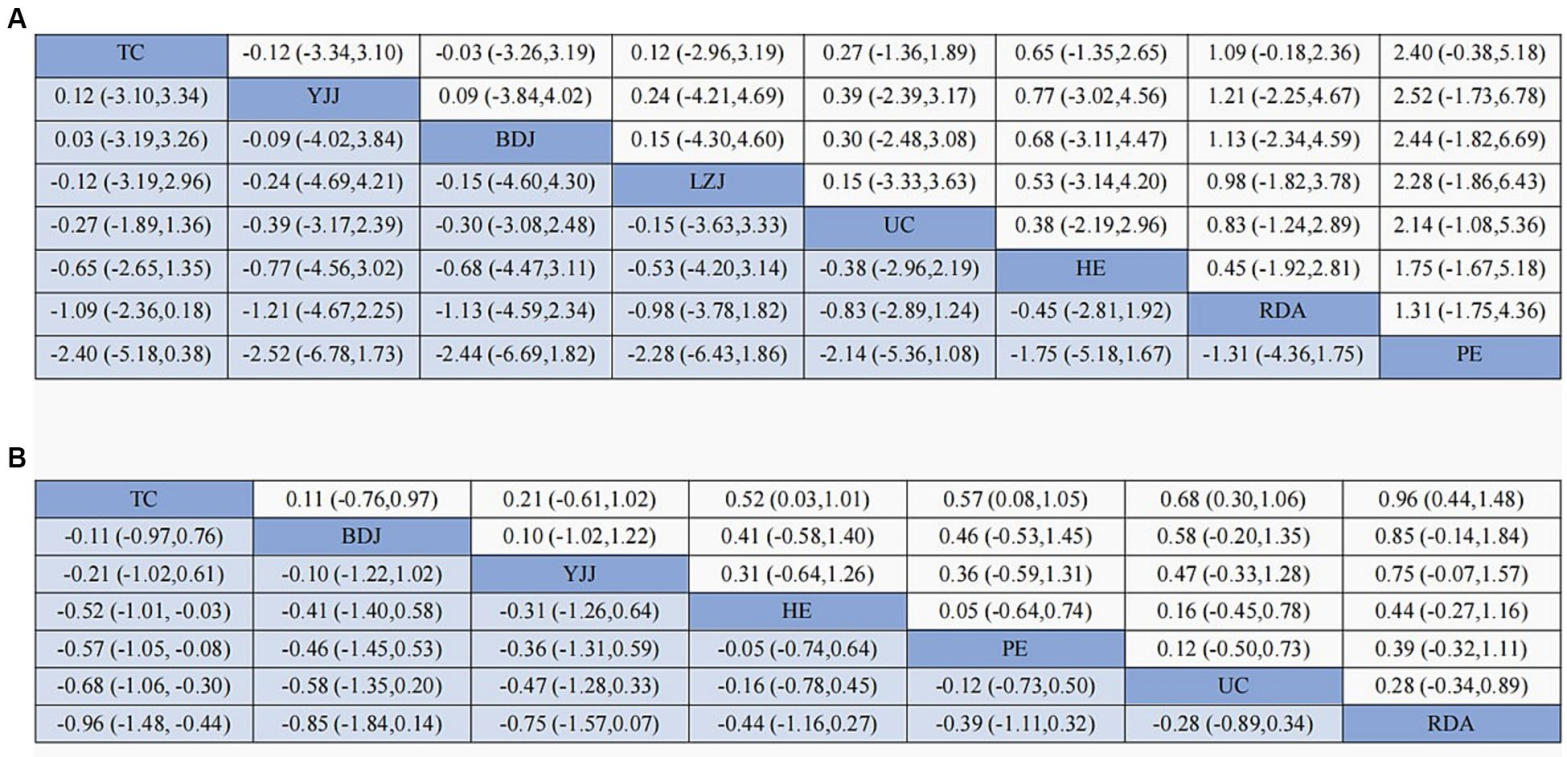
Results of the network meta-analysis (SMD vs. 95% CI) of the effects different TCM exercise on anxiety **(A)** and depression **(B)** in the adults.

**Figure 6:**
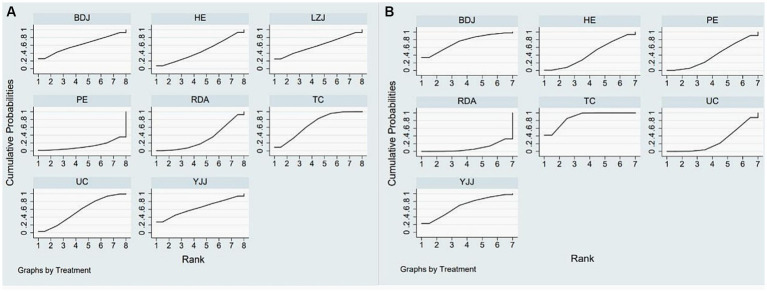
Anxiety **(A)** and depression **(B)** rank probability.

The results of the network meta-analysis are shown in Figure 5A, and compared with UC, Tai Chi (SMD: −0.27, 95% CI: −1.89, 1.46), Liu Zi Jue (SMD: −0.15, 95% CI: −3.63, 3.33), Yi Jin Jing (SMD: −0.39, 95% CI: −3.17, 2.39), Ba Duan Jin (SMD: −0.30, 95% CI: −3.08, 2.48), routine daily activities (SMD: 0.83, 95% CI: −1.24,2.89), health education (SMD: 0.38, 95% CI: −2.19,2.96), physical exercise (SMD: 2.14, 95% CI: −1.08,5.36) had better efficacy. As shown in the probability ranking table in Figure 6A, Tai Chi exercise had the highest probability of being the best intervention for anxiety in older adults (68.3%), followed by Yi Jin Jing (63.6%).

Figure 4B displays the network graph with depression as the outcome indicator, encompassing 27 studies and 7 interventions. The consistency model was utilized to compare different interventions following node segmentation and node split analysis. In Figure 5B, it is evident that compared to UC, Tai Chi (SMD: −0.68, 95% CI: −1.06, −0.30), Ba Duan Jin (SMD: −0.58, 95% CI: −1.35,0.20), Yi Jin Jing (SMD: −0.47, 95% CI: −1.28,0.33), routine daily activities (SMD: 0.28, 95% CI: −0.34,0.89), health education (SMD: −0.16, 95% CI: −0.78,0.45), and physical exercise (−0.12, 95% CI: −0.73,0.50) all showed better efficacy. Moreover, as indicated in the probability ranking table in Figure 6B, Tai Chi exercise demonstrated the highest likelihood of being the optimal intervention for depression among older adults (87.8%), followed by Ba Duan Jin (74.1%).

### Publication bias

**Figure 7:**
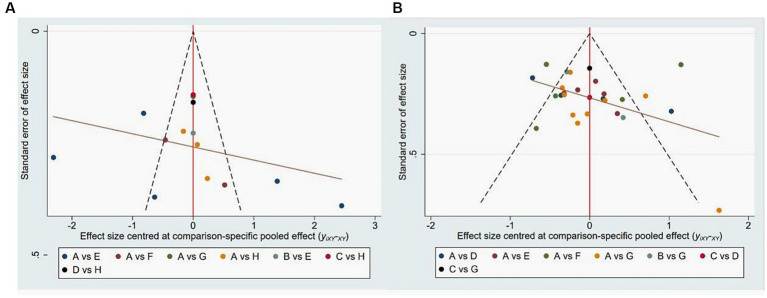
Publication bias of four Chinese traditional exercises on anxiety **(A)** and depression **(B)**. In the picture **(A)**, A is Tai Chi, B is Liu Zi Jue, C is Yi Jin Jing, D is Ba Duan Jin, E is routine daily activity, F is health education, G is physical activity, and H is usual care. In the picture **(B)**, A is Tai Chi, B is Ba Duan Jin, C is Yi Jin Jing, D is routine daily activity, E is health education, F is physical activity, and G is usual care.

We utilized anxiety and depression as outcome measures and created funnel plots using STATA software version 15.1. The results from the funnel plots for anxiety and depression (Figure 7) indicated that they were mainly symmetrical, suggesting the absence of publication bias.

### Sensitivity analysis

To assess the robustness of the results and investigate the heterogeneity contributed by each study, sensitivity analyses were performed by individually excluding each study. Subsequently, subgroup analyses were conducted to explore the impact of different study characteristics on the observed effects and identify potential sources of heterogeneity. The results showed that even after excluding any single study in the sensitivity analysis, the results remained robust.

## Discussion

As the global population continues to age, mental health significantly impacts the daily lives of older adults. The complex causes of anxiety and depression have made it difficult to develop interventions that are both effective and safe. In recent years, exercise therapy, including both active and passive activities, has gained recognition as an alternative approach to addressing these conditions. In China, the “Healthy China 2030” plan, issued by the Central Committee of the Communist Party of China, specifically aims to address common psychological issues such as anxiety and depression. It emphasizes the importance of strengthening psychological and behavioral interventions (Tan et al., 2019).

Recently, there has been a significant rise in the utilization of TCM exercise treatments. to address anxiety and depression among the older adult population. These therapies include practices such as Yi Jin Jing, Wu Qin Xi, Ba Duan Jin, and Tai Chi. As alternatives to pharmacological treatments, these four traditional exercise forms offer distinct philosophies and methodologies that differ from Western pharmaceutical approaches. They adopt a holistic perspective on the human body and prioritize achieving harmony through physical therapeutic techniques. By incorporating principles such as differential diagnosis and treatment, balancing the Yin-Yang aspects, and harmonizing deficiencies and excesses, these exercise modalities accurately represent the intricacies and transformational principles of TCM in promoting meridian flow and dialectical treatment (Cheng et al., 2023). TCM’s holistic perspective integrates various theories, including the Yin-Yang concept, health preservation, meridian theory, and Chinese philosophy, into a therapeutic framework known as “treating both the internal and external.” This strategy emphasizes the utilization of TCM exercises to enhance physical health and alleviate anxiety and depression.

Previous studies have not identified the most effective TCM intervention for treating depression and anxiety. Therefore, we evaluated the effectiveness of different TCM exercises for treating anxiety and depression in older adults using direct and indirect evidence. The included studies consisted of 30 randomized controlled trials that compared various types of TCM exercises to a control group. We analyzed anxiety and depression scores over treatment durations ranging from 3 weeks to 1 year. Our network meta-analysis ranked the SUCRA values for anxiety as follows: Tai Chi > Liu Zi Jue > Yi Jin Jing > Ba Duan Jin > routine daily activities > health education > physical exercise > usual care. The probability ranking for depression was: Tai Chi > Ba Duan Jin > Yi Jin Jing > routine daily activities > health education > physical exercise > usual care.

This study analyzed the improvement of non-pharmacological treatments for anxiety and depression in older adults, which is consistent with the results of previous traditional meta-analyses (Yin et al., 2021). The four types of traditional Chinese medicine exercises included in the study have all been shown to some extent to improve anxiety and depression in older adults, with tai chi being proven as the most beneficial intervention. Based on this, the study included four types of traditional Chinese medicine exercises as non-pharmacological treatment models, and further evaluated their therapeutic efficacy compared to traditional non-pharmacological treatments such as daily activities, health education, and sports activities. It is worth noting that we found tai chi to be effective in improving anxiety and depression in older adults.

Tai Chi, as a conventional healthcare modality, is a moderate-intensity aerobic fitness exercise that is deemed safe and dependable. It exerts beneficial effects on blood harmonization, cardiac function enhancement, and balance improvement, and is frequently employed in the management of chronic cardiovascular diseases, chronic lung diseases, and degenerative joint diseases (Gao et al., 2021; Wiedenmann et al., 2023). Typically comprising 12 or 24 postures, each lasting 30–60 min, Tai Chi has been the subject of numerous studies demonstrating its potential to ameliorate anxiety and depression, as well as enhance the quality of life in older adults (Qin et al., 2021; Wang et al., 2021; Zhao et al., 2021). This effect may be related to the reduced activity of the sympathetic nervous system caused by Tai Chi exercise. Researchers found that Tai Chi exercise can induce the production of certain special cytokines, such as transforming growth factor-β and interleukin-10, by measuring the levels of cortisol in saliva. The production of these cytokines helps improve quality of life and reduce psychological stress (Esch et al., 2007). Our findings are consistent with the results of previous systematic review studies, Tai Chi exercise when one of the effective ways to treat anxiety and depression. As a leisure activity, Tai Chi has the characteristics of safety, low exercise intensity, and not being affected by the venue, which can help older adults to reduce the weakening of physical and cognitive functions due to aging, the negative impact and economic burden, etc. (Wang et al., 2016). However, a randomized controlled study found that tai chi had a limited effect on depression in older adults. This may be because the control group engaged in simple physical exercises, while tai chi is more complex and harder for participants to accept. Additionally, in an unsupervised setting, participants found it difficult to consistently adhere to tai chi practice (Noradechanunt et al., 2017). Fortunately, this study considers Tai Chi as a potential non-pharmacological alternative for improving anxiety and depression in older adults. However, further clarification is needed as most participants in the experiments were aware of receiving Tai Chi intervention, which may lead to an expectancy effect when reporting anxiety and depression levels, resulting in a tendency to report better outcomes. This could potentially affect the objectivity of the experimental results. Additionally, the lack of blinding may introduce bias in the assessment of outcomes, thereby impacting the accuracy of the results. Taking these factors into consideration, it is necessary to establish the most effective treatment plan tailored to different levels of anxiety and depression, to develop more precise and individualized treatment strategies.

This study shows that Qigong (including Liu Zi Jue, Yi Jin Jing, and Ba Duan Jin) has an effect on anxiety and depression, which is consistent with the results of previous traditional meta-analyses (Yin and Dishman, 2014). Qigong, as a traditional Chinese medicine exercise modality, encompasses practices aimed at healthcare, maintenance of well-being, and disease elimination (Jahnke et al., 2010). It involves the integration of breath regulation, physical activity adjustment, and consciousness regulation (harmonizing breath, body, and mind) as a comprehensive approach to fortifying the body’s resilience, preventing and curing diseases, prolonging life, and developing potential (Chan et al., 2012). Mainly based on extreme abdominal breathing as the five viscera and six internal organs exercise method, can significantly enhance the cardiopulmonary function and digestion and absorption function, and make a person calm and quiet, mainly used in the treatment of chronic pulmonary diseases (Xiao et al., 2020; Gao et al., 2021). Therefore, its SUCRA values of anxiety and depression behind Tai Chi. The evidence from our research to date has found that Qigong may be less suitable for older people suffering from anxiety and depression compared to Tai Chi. However, this needs further confirmation.

Overall, our research has certain clinical significance. Firstly, our study provides evidence for the benefits of incorporating four types of traditional Chinese medicine exercises in alleviating anxiety and depression in older adults. By conducting a systematic review and analysis of different traditional Chinese medicine exercise interventions, our research offers valuable information for the use of these exercises as treatment options for anxiety and depression in older adults. Secondly, our study fills the research gap by comparing the clinical effectiveness of different traditional Chinese medicine exercise techniques in managing anxiety and depression in older adults. By comparing four different interventions of traditional Chinese medicine exercises, our research contributes to understanding the relative effectiveness of different forms of traditional Chinese medicine exercises in improving mental health outcomes. Lastly, our study utilizes traditional meta-analysis and network meta-analysis methods to analyze the data. The combination of these two methods allows for a more comprehensive evaluation of the included studies and a quantitative assessment of the effectiveness of traditional Chinese medicine exercise interventions. Therefore, these evidence-based findings can be recommended to clinical practitioners as more accurate non-pharmacological treatment options for older adults experiencing anxiety and depression.

Our study has several limitations. Firstly, significant heterogeneity was observed in the traditional meta-analysis, which may be due to variations in patients’ practice frequency, treatment duration, and daily habits. Additionally, the control group utilized different intervention measures, including other types of physical exercise, which may have already had some positive effects on physical activity. This could potentially mask the differences between the experimental and control groups, making the actual impact of physical exercise on anxiety and depression less clear. Moreover, the control group included individuals who did not engage in any exercise, which may contribute to higher levels of anxiety and depression, making the differences between the experimental and control groups more apparent. Fortuitously, the network meta-analysis did not reveal any inconsistencies or variations, indicating the absence of heterogeneity in the findings. However, in some of the included studies, the effectiveness of the treatment may have been compromised, which may have affected our findings. Second, due to the characteristics of TCM exercise therapy, the implementation of blinding participants is either unfeasible or challenging, thereby giving rise to potential biases in the study. Therefore, it is necessary to conduct large-scale RCTs using rigorous methods to test our findings. Nonetheless, our research continues to offer dependable data for making therapeutic decisions in geriatric individuals afflicted by anxiety and depression. Moreover, it is imperative to exercise caution when interpreting the findings of this study, primarily attributable to the paucity of research studies concerning qigong (Ba Duan Jin, Wu Qin Xi, Yi Jin Jing, Liu Zi Jue) and the limitations of SUCRA. Therefore, it is necessary to increase more randomized controlled studies on Qigong. In addition, there are various traditional Chinese forms of exercise. This network meta-analysis only includes Tai Chi, Ba Duan Jin, Yi Jin Jing, and Liu Zi Jue for analysis, and it only focuses on older participants. Therefore, caution should be exercised when generalizing this conclusion to other populations, and further research is needed to validate the results.

In forthcoming research endeavors, there is a paramount necessity to enhance the objectivity, standardization, and overall quality of research trial designs. To improve the reliability and effectiveness of research, the method of double-blind randomized controlled trials can be adopted to reduce intervention effects and biases. Whenever feasible, a direct comparison between Tai Chi and different Qigong practices should be prioritized to minimize the risk of bias in indirect comparison studies and to scientifically select non-pharmacological treatment approaches that yield better clinical outcomes.

## Conclusion

The results of the Network Meta-analysis show that TCM exercise therapy can improve anxiety and depression in older adults. Among the four TCM exercise therapies included, Tai Chi may be a preferred option for treating anxiety and depression in older adults. Nevertheless, further research is required to validate the effectiveness of this exercise therapy through larger and more rigorous clinical trials.

## Author contributions

YD: Data curation, Methodology, Software, Validation, Writing – original draft. JY: Formal analysis, Project administration, Supervision, Writing – review & editing. XK: Data curation, Methodology, Software, Validation, Writing – original draft. XZ: Project administration, Supervision, Writing – review & editing. LD: Data curation, Methodology, Software, Writing – original draft. GC: Data curation, Methodology, Software, Writing – original draft. JQ: Project administration, Supervision, Writing – review & editing.

## References

[ref1] Akbari A. Mirakhori F. Ashouri M. Tehrani N. N. (2022). The effect of micronutrient intake on cognitive function and physical activity of the elderly. Int J Sport Stud Health 4:360. doi: 10.5812/intjssh.121360

[ref2] Bonab S. B. Parvaneh M. (2022). The effect of twelve weeks of tai chi exercises on sleep quality, pain perception, and death anxiety in elderly women. Ann. Med. Psychol. 180, 905–911. doi: 10.1016/j.amp.2022.01.019

[ref3] Borenstein M. Hedges L. V. Higgins J. P. T. Rothstein H. R. (2010). A basic introduction to fixed-effect and random-effects models for meta-analysis. Res. Synth. Methods 1, 97–111. doi: 10.1002/jrsm.12, PMID: 26061376

[ref4] Cassiano A. D. N. Da Silva T. S. Do Nascimento C. Q. Wanderley E. M. Prado E. S. De Morais Santos T. M. . (2020). Effects of physical exercise on cardiovascular risk and quality of life in hypertensive elderly people. Cien. Saude Colet. 25, 2203–2212. doi: 10.1590/1413-81232020256.27832018, PMID: 32520265

[ref5] Chan C. L. W. Wang C.-W. Ho R. T. H. Ng S.-M. Ziea E. T. C. Wong V. T. (2012). Qigong exercise for the treatment of fibromyalgia: A systematic review of randomized controlled trials. J. Altern. Complement. Med. 18, 641–646. doi: 10.1089/acm.2011.0347 22757663

[ref6] Cheng M. Wang Y. Wang S. Cao W. Wang X. (2023). Network meta-analysis of the efficacy of four traditional Chinese physical exercise therapies on the prevention of falls in the elderly. Front. Public Health 10:599. doi: 10.3389/fpubh.2022.1096599, PMID: 36684937 PMC9846771

[ref7] Chou K.-L. Lee P. W. H. Yu E. C. S. Macfarlane D. Cheng Y.-H. Chan S. S. C. . (2004). Effect of tai Chi on depressive symptoms amongst Chinese older patients with depressive disorders: a randomized clinical trial. Int. J. Geriatr. Psychiatry 19, 1105–1107. doi: 10.1002/gps.1178, PMID: 15497192

[ref8] Esch T. Duckstein J. Welke J. Braun V. (2007). Mind/body techniques for physiological and psychological stress reduction: stress management via tai Chi training—a pilot study. Med. Sci. Monit. 13:CR488. PMID: 17968296

[ref9] Faraji F. Nouhi S. Peiade-koohsar A. Janbozorgi M. (2021a). The effectiveness of god-inclined spiritually multidimensional psychotherapy (SMP) in improving symptoms of psychological disorders and post-traumatic stress disorder. J Assess Res Appl Counsel 3, 1–16. doi: 10.52547/jarcp.3.1.1

[ref10] Faraji F. Nouhi S. Peiade-koohsar A. Janbozorgi M. (2021b). The effects of short-term intensive scanning psychotherapy on defense styles and improving the quality of life of addicts under methadone maintenance treatment (MMT). J Assess Res Appl Counsel 2, 61–72. doi: 10.52547/jarcp.2.4.61

[ref11] Gao L. (2016). Study on the effect of fitness qigong intervention on anxiety and depressive symptoms in elderly people. J Shandong Instit Phys Educ 32, 56–59. doi: 10.14104/j.cnki.1006-2076.2016.01.011(In China)

[ref12] Gao P. Tang F. Liu W. He K. Mo Y. (2021). Effect of liuzijue qigong on patients with stable chronic obstructive pulmonary disease A systematic review and meta-analysis. Medicine 100:e27344. doi: 10.1097/md.0000000000027344, PMID: 34731105 PMC8519198

[ref13] Gao Y. Yu L. Li X. Yang C. Wang A. Huang H. (2021). The effect of different traditional Chinese exercises on blood lipid in middle-aged and elderly individuals: A systematic review and network Meta-analysis. Life 11:70714. doi: 10.3390/life11070714, PMID: 34357085 PMC8305451

[ref14] Ge Y. Liu H. Wu Q. Chen A. Gao Z. Xing F. . (2022). Effects of a short eight tai Chi-forms for the pre-frail elderly people in senior living communities. Physiother. Theory Pract. 38, 1928–1936. doi: 10.1080/09593985.2021.1926023, PMID: 34076569

[ref15] Hay S. I. Jayaraman S. P. Truelsen T. Sorensen R. J. D. Millear A. Giussani G. . (2017). GBD 2015 disease and injury incidence and prevalence collaborators. Global, regional, and national incidence, prevalence, and years lived with disability for 310 diseases and injuries, 1990-2015: a systematic analysis for the global burden of disease study 2015. Lancet 388:1545. doi: 10.1016/S0140-6736(16)31678-6 PMC5055577 27733282

[ref16] Hong S. Zhang J. Liu X. (2017). The effect of taijiquan practice on the physical and mental health of middle-aged and elderly people. Chin. J. Gerontol. 37, 2010–2011.

[ref17] Hsu C.-Y. Moyle W. Cooke M. Jones C. (2016). Seated tai Chi versus usual activities in older people using wheelchairs: A randomized controlled trial. Complement. Ther. Med. 24, 1–6. doi: 10.1016/j.ctim.2015.11.006, PMID: 26860794

[ref18] Huang N. Li W. Rong X. Champ M. Wei L. Li M. . (2019). Effects of a modified tai Chi program on older people with mild dementia: A randomized controlled trial. J. Alzheimers Dis. 72, 947–956. doi: 10.3233/jad-190487, PMID: 31743998

[ref19] Irwin M. R. Olmstead R. Carrillo C. Sadeghi N. Breen E. C. Witarama T. . (2014). Cognitive behavioral therapy vs. tai Chi for late life insomnia and inflammatory risk: A randomized controlled comparative efficacy trial. Sleep 37, 1543–1552. doi: 10.5665/sleep.4008, PMID: 25142571 PMC4153053

[ref20] Jaeschke K. Hanna F. Ali S. Chowdhary N. Dua T. Charlson F. (2021). Global estimates of service coverage for severe mental disorders: findings from the WHO mental health atlas 2017. Glob Mental Health 8:e27. doi: 10.1017/gmh.2021.19, PMID: 34367650 PMC8320004

[ref21] Jahnke R. A. Larkey L. K. Rogers C. (2010). Dissemination and benefits of a replicable tai Chi and Qigong program for older adults. Geriatr. Nurs. 31, 272–280. doi: 10.1016/j.gerinurse.2010.04.012, PMID: 20682405

[ref22] Kan A. (2008). Health implications of participation in Taijiquan exercise for older adults. Chin. J. Health Psychol. 4, 477–478. doi: 10.13342/j.cnki.cjhp.2008.04.054

[ref23] Krause K. R. Chung S. Adewuya A. O. Albano A. M. Babins-Wagner R. Birkinshaw L. . (2021). International consensus on a standard set of outcome measures for child and youth anxiety, depression, obsessive-compulsive disorder, and post-traumatic stress disorder. Lancet Psychiatry 8, 76–86. doi: 10.1016/S2215-0366(20)30356-4, PMID: 33341172

[ref24] Lam L. C. W. Chan W. M. Kwok T. C. Y. Chiu H. F. K. (2014). Effectiveness of tai Chi in maintenance of cognitive and functional abilities in mild cognitive impairment: a randomised controlled trial. Hong Kong Med. J. 20, 20–23. PMID: 25001031

[ref25] Lavretsky H. Alstein L. L. Olmstead R. E. Ercoli L. M. Riparetti-Brown M. Cyr N. S. . (2011). Complementary use of tai Chi Chih augments escitalopram treatment of geriatric depression: A randomized controlled trial. Am. J. Geriatr. Psychiatry 19, 839–850. doi: 10.1097/JGP.0b013e31820ee9ef, PMID: 21358389 PMC3136557

[ref26] Lei H. Ma Z. Tian K. Liu K. Wang J. Zhu X. . (2022). The effects of different types of tai Chi exercises on motor function in patients with Parkinson's disease: A network meta-analysis. Front. Aging Neurosci. 14:6027. doi: 10.3389/fnagi.2022.936027, PMID: 36105909 PMC9465240

[ref27] Leung R. W. M. McKeough Z. J. Peters M. J. Alison J. A. (2013). Short-form Sun-style t'ai chi as an exercise training modality in people with COPD. Eur. Respir. J. 41, 1051–1057. doi: 10.1183/09031936.00036912, PMID: 22878879

[ref28] Li Y. Yang T. Zhou H. Cao J. (2017). Evaluation of the effect of long-term tai chi exercise in the consolidation treatment of cured elderly with anxiety disorders. Chin. J. Gerontol. 37, 1992–1994.

[ref29] Li Y. Zhang H. Wang Y. (2019). Tai Chi ameliorates coronary heart disease by affecting serum levels of miR-24 and miR-155. Front. Physiol. 10:587. doi: 10.3389/fphys.2019.00587, PMID: 31191331 PMC6548805

[ref30] Liang C. Gao C. Zhang J. Ye Q. Zhai L. Zhao F. . (2019). Traditional Chinese medicine training for cardiac rehabilitation: a randomized comparison with aerobic and resistance training. Coron. Artery Dis. 30, 360–366. doi: 10.1097/mca.0000000000000734, PMID: 31107694

[ref31] Liao J. (2015). Effects of 24w tai Chi exercise on the mental health of urban elderly women. Chin. J. Gerontol. 35, 7232–7233.

[ref32] Liao S. J. Tan M. P. Chong M. C. Chua Y. P. (2018). The impact of combined music and tai Chi on depressive symptoms among community-dwelling older persons: A cluster randomized controlled trial. Issues Ment. Health Nurs. 39, 398–402. doi: 10.1080/01612840.2017.1417519, PMID: 29436896

[ref33] Liu Q. (2016). The effects of 16 weeks of tai chi exercise and 8 weeks of cessation on the mental health of elderly women. J Shand Instit Phys Educ 32, 99–103. doi: 10.14104/j.cnki.1006-2076.2016.06.018

[ref34] Liu Y. Beliveau A. Wei Y. Chen M. Y. Record-Lemon R. Kuo P.-L. . (2022). A gentle introduction to Bayesian network Meta-analysis using an automated R package. Multivar. Behav. Res. 58, 706–722. doi: 10.1080/00273171.2022.2115965, PMID: 36254763

[ref35] Liu J. Tao J. Xia R. Li M. Huang M. Li S. . (2021). Mind-body exercise modulates locus Coeruleus and ventral tegmental area functional connectivity in individuals with mild cognitive impairment. Front. Aging Neurosci. 13:6807. doi: 10.3389/fnagi.2021.646807, PMID: 34194314 PMC8236862

[ref36] Liu J. Xie H. Liu M. Wang Z. Zou L. Yeung A. S. . (2018). The effects of tai Chi on heart rate variability in older Chinese individuals with depression. Int. J. Environ. Res. Public Health 15:2771. doi: 10.3390/ijerph15122771, PMID: 30544491 PMC6313592

[ref37] Ma C. Zhou W. Tang Q. Huang S. (2018). The impact of group-based tai chi on health-status outcomes among community-dwelling older adults with hypertension. Heart Lung 47, 337–344. doi: 10.1016/j.hrtlng.2018.04.007, PMID: 29778251

[ref38] Muhammad F. (2020). Exercise: A free medicine of all time. Int J Sport Stud Health 3:4519. doi: 10.5812/intjssh.104519

[ref39] Noradechanunt C. Worsley A. Groeller H. (2017). Thai yoga improves physical function and well-being in older adults: A randomised controlled trial. J. Sci. Med. Sport 20, 494–501. doi: 10.1016/j.jsams.2016.10.007, PMID: 27866841

[ref40] Peng M. Mo B. Liu Y. Xu M. Song X. Liu L. . (2020). Prevalence, risk factors and clinical correlates of depression in quarantined population during the COVID-19 outbreak. J. Affect. Disord. 275, 119–124. doi: 10.1016/j.jad.2020.06.035, PMID: 32658813 PMC7330582

[ref41] Qin J. Chen Y. Guo S. You Y. Xu Y. Wu J. . (2021). Effect of tai Chi on quality of life, body mass index, and waist-hip ratio in patients with type 2 diabetes mellitus: A systematic review and Meta-analysis. Front. Endocrinol. 11:543627. doi: 10.3389/fendo.2020.543627, PMID: 33542702 PMC7851054

[ref42] Redwine L. S. Pung M. A. Wilson K. Bangen K. J. Delano-Wood L. Hurwitz B. (2020). An exploratory randomized sub-study of light-to-moderate intensity exercise on cognitive function, depression symptoms and inflammation in older adults with heart failure. J. Psychosom. Res. 128:109883. doi: 10.1016/j.jpsychores.2019.109883, PMID: 31786338 PMC7571258

[ref43] Salanti G. Ades A. E. Ioannidis J. P. A. (2011). Graphical methods and numerical summaries for presenting results from multiple-treatment meta-analysis: an overview and tutorial. J. Clin. Epidemiol. 64, 163–171. doi: 10.1016/j.jclinepi.2010.03.016, PMID: 20688472

[ref44] Santomauro D. F. Herrera A. M. M. Shadid J. Zheng P. Ashbaugh C. Pigott D. M. . (2021). Global prevalence and burden of depressive and anxiety disorders in 204 countries and territories in 2020 due to the COVID-19 pandemic. Lancet 398, 1700–1712. doi: 10.1016/s0140-6736(21)02143-7, PMID: 34634250 PMC8500697

[ref45] Shim S. Yoon B.-H. Shin I.-S. Bae J.-M. (2017). Network meta-analysis: application and practice using Stata. Epidemiol Health 39:e2017047. doi: 10.4178/epih.e2017047, PMID: 29092392 PMC5733388

[ref46] Silva R. Rufino C. Galvão L. Vancini R. L. Santos D. A. T. de Lira C. . (2022). Motivation for Brazilian older adult women to join a community physical activity program before COVID-19 pandemic. Int J Sport Stud Health 5:128560. doi: 10.5812/intjssh-128560

[ref47] Solianik R. Mickeviciene D. Zlibinaite L. Cekanauskaite A. (2021). Tai chi improves psychoemotional state, cognition, and motor learning in older adults during the COVID-19 pandemic. Exp. Gerontol. 150:111363. doi: 10.1016/j.exger.2021.111363, PMID: 33887380 PMC8054611

[ref48] Song J. Wei L. Cheng K. Lin Q. Xia P. Wang X. . (2022). The effect of modified tai Chi exercises on the physical function and quality of life in elderly women with knee osteoarthritis. Front. Aging Neurosci. 14:860762. doi: 10.3389/fnagi.2022.860762, PMID: 35721018 PMC9204295

[ref49] Soylu Y. Arslan E. Kilit B. (2022). Psychophysiological responses and cognitive performance: A systematic review of mental fatigue on soccer performance. Int J Sport Stud Health 4:244. doi: 10.5812/intjssh.124244

[ref50] Su K. Yuan J. Liu H. Luo M. Li Q. Liu S. . (2022). The comparative effectiveness of traditional Chinese medicine exercise therapies in elderly people with mild cognitive impairment: A systematic review and network Meta-analysis. Front. Neurol. 13:5190. doi: 10.3389/fneur.2022.775190, PMID: 35370918 PMC8966650

[ref51] Sun P. Fang L. Qi R. Chen X. Yan J. (2022). Study on the clinical efficacy and brain network mechanism of modified Yi Jin Jing in patients with mild depression after stroke. Chin J Rehabil Med 37, 1506–1510.

[ref52] Taheri M. Irandost K. Mirmoezzi M. Ramshini M. (2018). Effect of aerobic exercise and Omega-3 supplementation on psychological aspects and sleep quality in prediabetes elderly women. Sleep Hypn 21, 170–174. doi: 10.5350/Sleep.Hypn.2019.21.0185

[ref53] Tan X. Zhang Y. Shao H. (2019). Healthy China 2030, a breakthrough for improving health. Glob. Health Promot. 26, 96–99. doi: 10.1177/1757975917743533, PMID: 29297762

[ref54] Tsang H. W. H. Tsang W. W. N. Jones A. Y. M. Fung K. M. T. Chan A. H. L. Chan E. P. . (2013). Psycho-physical and neurophysiological effects of qigong on depressed elders with chronic illness. Aging Ment. Health 17, 336–348. doi: 10.1080/13607863.2012.732035, PMID: 23072658

[ref55] Wang H. Wei A. Lu Y. Yu B. Chen W. Lu Y. . (2016). Simplified tai Chi program training versus traditional tai Chi on the functional movement screening in older adults. Evid. Based Complement. Alternat. Med. 2016, 1–6. doi: 10.1155/2016/5867810, PMID: 27956920 PMC5124480

[ref56] Wang L.-C. Ye M.-Z. Xiong J. Wang X.-Q. Wu J.-W. Zheng G.-H. (2021). Optimal exercise parameters of tai chi for balance performance in older adults: A meta-analysis. J. Am. Geriatr. Soc. 69, 2000–2010. doi: 10.1111/jgs.17094, PMID: 33769556

[ref57] Wei C. (2013). The impact of Baduanjin on the mental health of older adults in urban communities. Chin. J. Gerontol. 33, 3472–3473.

[ref58] Wiedenmann T. Held S. Rappelt L. Grauduszus M. Spickermann S. Donath L. (2023). Exercise based reduction of falls in communitydwelling older adults: a network meta-analysis. Eur. Rev. Aging Phys. Act. 20:1. doi: 10.1186/s11556-023-00311-w, PMID: 36707758 PMC9883964

[ref59] Wu C.-S. Yu S.-H. Lee C.-Y. Tseng H.-Y. Chiu Y.-F. Hsiung C. A. (2017). Prevalence of and risk factors for minor and major depression among community-dwelling older adults in Taiwan. Int. Psychogeriatr. 29, 1113–1121. doi: 10.1017/s1041610217000199, PMID: 28390440

[ref60] Xiao L. Duan H. Li P. Wu W. Shan C. Liu X. (2020). A systematic review and meta-analysis of Liuzijue in stable patients with chronic obstructive pulmonary disease. BMC Complement Med Ther 20:308. doi: 10.1186/s12906-020-03104-1, PMID: 33054800 PMC7557061

[ref61] Yeh G. Y. Litrownik D. Wayne P. M. Beach D. Klings E. S. Reyes Nieva H. . (2020). BEAM study (breathing, education, awareness, movement): a randomised controlled feasibility trial of tai chi exercise in patients with COPD. BMJ Open Respir. Res. 7:697. doi: 10.1136/bmjresp-2020-000697, PMID: 33219007 PMC7682460

[ref62] Yildirim P. Ofluoglu D. Aydogan S. Akyuz G. (2016). Tai Chi vs. combined exercise prescription: A comparison of their effects on factors related to falls. J. Back Musculoskelet. Rehabil. 29, 493–501. doi: 10.3233/bmr-150645, PMID: 26519119

[ref63] Yin J. Dishman R. K. (2014). The effect of tai Chi and Qigong practice on depression and anxiety symptoms: A systematic review and meta-regression analysis of randomized controlled trials. Ment. Health Phys. Act. 7, 135–146. doi: 10.1016/j.mhpa.2014.08.001

[ref64] Yin J. Tang L. Dishman R. K. (2021). The effects of a single session of mindful exercise on anxiety: A systematic review and meta-analysis. Ment. Health Phys. Act. 21:100403. doi: 10.1016/j.mhpa.2021.100403

[ref65] Yuan L. Zhang H. Zhou F. Xue X. Fang S. (2016). Improvement effects of tai chi on depressive state, sleep quality and quality of life in elderly patients with chronic congestive heart failure with depressive state. Guangxi Med 38, 1547–1550.

[ref66] Zenebe Y. Akele B. Selassie M. W. Necho M. (2021). Prevalence and determinants of depression among old age: a systematic review and meta-analysis. Ann. Gen. Psychiatry 20:55. doi: 10.1186/s12991-021-00375-x, PMID: 34922595 PMC8684627

[ref67] Zhao J. Chau J. P. C. Lo S. H. S. Choi K. C. Liang S. (2021). The effects of sitting tai Chi on physical and psychosocial health outcomes among individuals with impaired physical mobility: A systematic review and meta-analysis. Int. J. Nurs. Stud. 118:103911. doi: 10.1016/j.ijnurstu.2021.103911, PMID: 33751992

[ref68] Zhong Z. Zhang W. Wan Z. (2006). The effect of fitness qigong Yi Jin Jing on the mental health status of elderly people. Chin Sci Behav Med 9, 850–851.

