# Supplementary material for: Exploring the efficacy of traditional Chinese medicine exercise in alleviating anxiety and depression in older adults: a comprehensive study with randomized controlled trial and network meta-analysis
Source: Front Psychol. 2023 Dec 11;14:1290471. doi: 10.3389/fpsyg.2023.1290471 (PMC10749367; doi:10.3389/fpsyg.2023.1290471)
Supplement: Supplementary file 1 [file Data_Sheet_1.docx]

Supplementary Material

Search strategy

**Cochrane**

| Step | Search strategy |
| --- | --- |
| #1 | MeSH descriptor: [Aged] explode all trees |
| #2 | (elderly):ti,ab,kw |
| #3 | #1 OR #2 |
| #4 | MeSH descriptor: [Tai Ji] explode all trees |
| #5 | (Chi, Tai):ti,ab,kw OR (Taijiquan):ti,ab,kw OR (Taiji):ti,ab,kw OR (T'ai Chi):ti,ab,kw OR (Tai Chi):ti,ab,kw OR (Ji Quan, Tai):ti,ab,kw OR (Tai Chi Chuan):ti,ab,kw OR (Tai Ji Quan):ti,ab,kw OR (Quan, Tai Ji):ti,ab,kw OR (Tai-ji):ti,ab,kw |
| #6 | MeSH descriptor: [Qigong] explode all trees |
| #7 | (Ch'i Kung):ti,ab,kw OR (Qi Gong):ti,ab,kw |
| #8 | (Liu Zi Jue):ti,ab,kw OR (Wu Qin Xi):ti,ab,kw OR (Six Healing Sounds):ti,ab,kw OR (Ba Duan Jin):ti,ab,kw OR (Traditional Chinese exercise):ti,ab,kw OR (Yi Jin Jing):ti,ab,kw |
| #9 | #4 OR #5 OR #6 OR #7 OR #8 |
| #10 | (randomized controlled trial):pt OR (controlled clinical trial):pt OR (randomized):ti,ab,kw |
| #11 | #3 AND #9 AND #10 |

**Web of science**

| Step | Search strategy |
| --- | --- |
| #1 | (TS=(Aged)) OR TS=(elderly) |
| #2 | ((((((((((((((((((TS=(Tai Ji)) OR TS=(Tai-ji)) OR TS=(Tai Chi)) OR TS=(Chi, Tai))) OR TS=(Tai Ji Quan)) OR TS=(Ji Quan, Tai)) OR TS=(Quan, Tai Ji)) OR TS=(Taiji)) OR TS=(Taijiquan)) OR TS=(T'ai Chi)) OR TS=(Tai Chi Chuan)) OR TS=(qigong))) OR TS=(Liu Zi Jue)) OR TS=(Wu Qin Xi)) OR TS=(Six Healing Sounds)) OR TS=(Ba Duan Jin)) OR TS=(Traditional Chinese exercise) OR TS=(Yi Jin Jing) |
| #3 | ((TS=(randomized controlled trial)) OR TS=(controlled clinical trial)) OR TS=(randomized) |
| #4 | #4 #1 AND #2 AND #3 |

**CNKI**

| Step | Search strategy |
| --- | --- |
| #1 | (SU % '太极' OR SU % '太极拳' OR SU % '中国传统运动' OR SU % '五禽戏' OR SU % '易筋经' OR SU % '八段锦' OR SU %'六字诀' OR SU % '气功') AND (SU % '老年人' OR SU % '老人' OR SU % '年老者') |

**VIP**

| Step | Search strategy |
| --- | --- |
| #1 | (M=太极 OR M=太极拳 OR M=五禽戏 OR M=八段锦 OR M=易筋经 OR M=六字诀 OR M=气功 OR M=中国传统运动) AND (M=老年人 OR M=老人 OR M=年老者) |

**Wang Fang**

| Step | Search strategy |
| --- | --- |
| #1 | (题名或关键词:(太极拳) or 题名或关键词:(太极) or 题名或关键词:(八段锦) or 题名或关键词:(五禽戏) or 题名或关键词:(易筋经) or 题名或关键词:(六字诀) or 题名或关键词:(气功) or 题名或关键词:(中国传统运动)) and (题名或关键词:(老年) or 题名或关键词:(老年人) or 题名或关键词:(年老者)) |


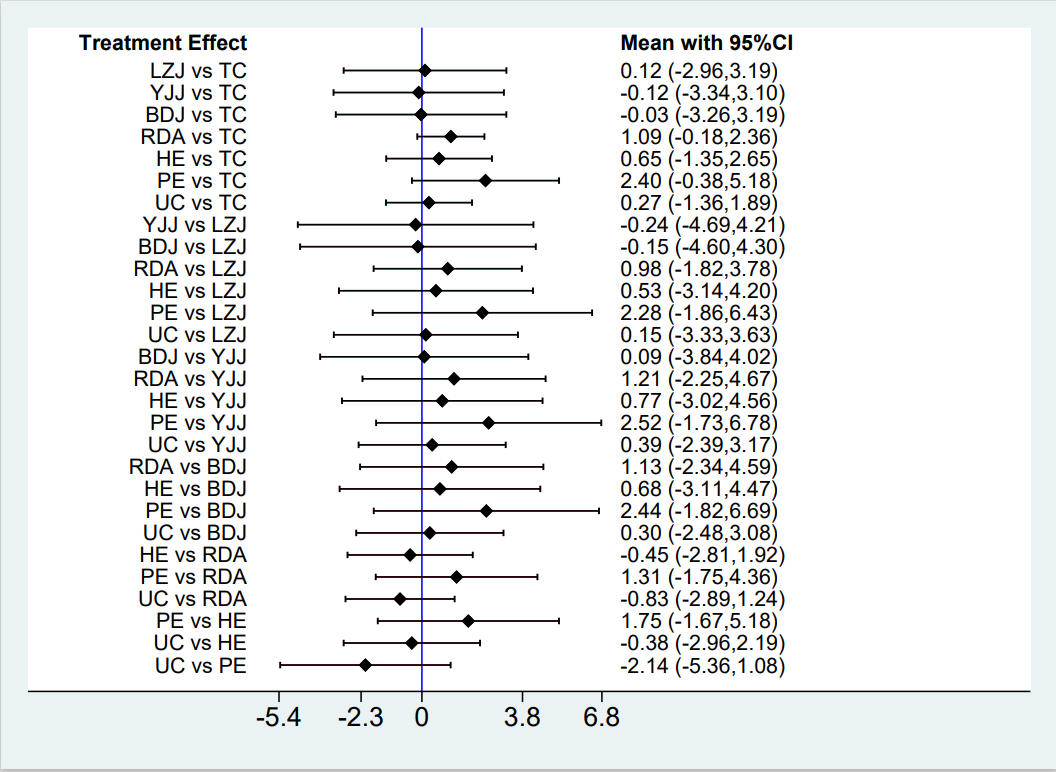


Supplementary Figure 1. Interval plot of network meta-analysis for anxiety in the elder


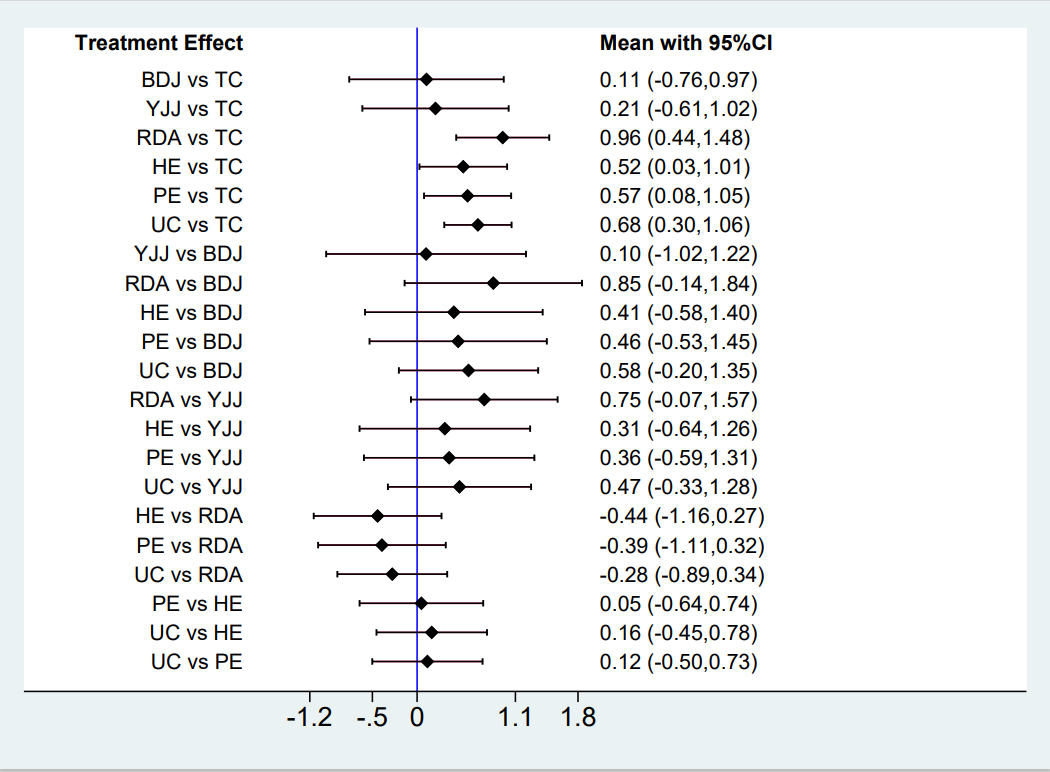


Supplementary Figure 2. Interval plot of network meta-analysis for depression in the elder
